# Atypical Electrophysiological Pattern in Hypokalemic Periodic Paralysis With CACNA1S Mutation: A Case Report

**DOI:** 10.7759/cureus.79539

**Published:** 2025-02-24

**Authors:** Kamal Haddouali, Hajar El Omari, Hicham El Otmani, Bouchra El Moutawakil, Mohammed Abdoh Rafai

**Affiliations:** 1 Research Laboratory on Diseases of the Nervous System, Neurosensory and Handicap, Hassan II University, Faculty of Medicine and Pharmacy, Casablanca, MAR; 2 Department of Neurology and Neurophysiological Explorations, Ibn Rochd University Hospital - Faculty of Medicine and Pharmacy, Casablanca, MAR; 3 Department of Neurology and Neurophysiological Explorations, Ibn Rochd University Hospital - Hassan II University, Faculty of Medicine and Pharmacy, Casablanca, MAR; 4 Genetics Laboratory, Faculty of Medicine and Pharmacy, Casablanca, MAR; 5 Research Laboratory on Diseases of the Nervous System, Neurosensory and Handicap, Faculty of Medicine and Pharmacy, Casablanca, MAR

**Keywords:** cacna1s, calcium channel, channelopathy, long exercise test, periodic paralysis

## Abstract

Hypokalemic periodic paralysis type 1 (PPHy-1) is a rare autosomal dominant disorder caused by mutations in the CACNA1S gene, leading to recurrent muscle weakness associated with hypokalemia. We describe a 19-year-old male presenting with recurrent episodes of muscle weakness lasting from 2 to 48 hours. During an attack, clinical examination revealed tetraparesis with axial motor deficits, but no bulbar or facial involvement. Serum potassium levels were 2.5 mmol/L, and nerve conduction studies (NCS) showed asymmetrical decrement in motor amplitudes. A long exercise test (LET) post-attack revealed pattern IV, an atypical finding for PPHy-1. Whole exome sequencing confirmed a heterozygous mutation in the *CACNA1S* gene, establishing the diagnosis. The patient responded well to oral potassium supplementation and prophylactic acetazolamide, which reduced the frequency of attacks. This case highlights the importance of integrating clinical, electrophysiological, and genetic findings in diagnosing PPHy-1. The unusual LET pattern IV suggests variability in the expression of PPHy-1, warranting further investigation into the variability of LET patterns in this disorder.

## Introduction

Hypokalemic periodic paralysis type 1 (PPHy-1) is a rare genetic disorder (1/100,000 in Europe) characterized by recurrent episodes of muscle weakness associated with hypokalemia [1]. This autosomal dominant condition is usually linked to mutations in the *CACNA1S* gene, which encodes the alpha-1 subunit of the L-type voltage-gated calcium channel. Electroneuromyography plays a crucial role in diagnosing periodic paralyses, and guiding genetic studies based on the patterns observed during long exercise tests (LET) [2]. We report a case of PPHy-1 in a patient who exhibited an unexpected pattern IV in the LET and achieved good control of attacks with oral acetazolamide.

## Case presentation

A 19-year-old Moroccan male patient, with no significant medical or family history, presented with recurrent episodes of muscle weakness in all four limbs, lasting from 2 to 48 hours, with spontaneous resolution. The first episode occurred at age 14, following intense physical exertion during a football match. A week later, a second attack occurred after the consumption of a large and unusual quantity of sweets. Subsequently, the patient experienced one to two attacks per month, triggered by intense physical exertion, a diet rich in carbohydrates, and exposure to cold. He was admitted to our department four years later due to a severe paralytic attack. Clinical examination revealed symmetrical proximo-distal tetraparesis (Medical Research Council: 2/5), associated with an axial motor deficit preventing the start position, walking, and bed transfers, without involvement of the face or bulbar muscles. Tendon reflexes, sensory examination, and muscle trophicity were normal, with no signs of myotonia or dysmorphic syndrome. There were no other systemic signs, and intellectual faculties were normal. Nerve conduction studies (NCS) during the attack revealed an asymmetrical decrement in motor amplitude across all four limbs, returning to normal values after the attack (Table 1). Distal motor latency, conduction velocity, F waves, and needle detection were normal.

**Table 1:** Nerve conduction studies’ features. There is a decrease in motor amplitudes in all four limbs with preservation of distal latencies and conduction velocities. Sensory potentials (amplitudes and velocities) are normal in all four limbs.

Nerve conduction studies				
	Distal latency (ms)	Conduction velocity (ms)	Amplitude during attack paralysis (mV for motor responses, µV for sensory responses)	Amplitude after attack paralysis (mV for motor responses, µV for sensory responses)
Motor nerves				
Right median	2.2	60.8	2.3	9.8
Left median	2.6	70	0.36	11.3
Right ulnar	2.1	71.1	4.4	8.2
Left ulnar	2.1	75.3	5.8	7.7
Right tibial	5.2	46.5	1.4	2.8
Left tibial	5.7	55.3	1.1	3.9
Right peroneal	3.2	53.7	1.8	2.9
Left peroneal	3.9	49	2.5	2.9
Sensory nerves				
Right median	1.1	81.8	60	72.3
Left median	1.2	50.9	45.9	58.3
Right ulnar	0.9	88.9	40.2	43.8
Left ulnar	1.1	72.7	28.8	27.7
Right sural	1.8	50	19.3	20
Left sural	1.5	54.3	19	19.3

**Figure 1:**
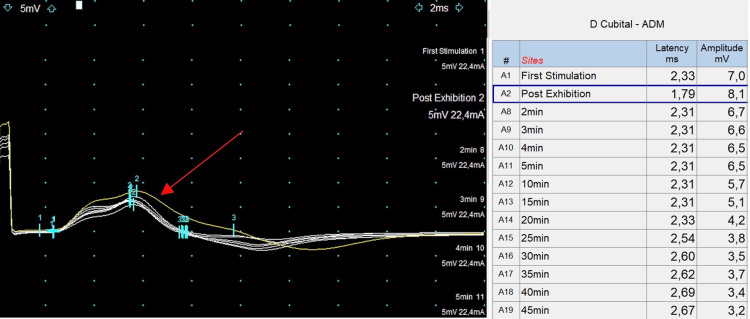
The long exercise test. The test shows a 12% increase in motor amplitude post-exercise (yellow motor response; red arrow) of the right cubital nerve, followed by a progressive decrease (-54% at the 45-minute mark), defining pattern IV.

The LET (McManis et al.) [3], performed after the resolution of the paralytic attack, revealed pattern IV, suggestive of hyperkalemic periodic paralysis (Figure 1).

However, serum potassium levels were hypokalemic at 2.5 mmol/L. The ECG and biological assessment (blood count, thyroid-stimulating hormone, renal function, blood and urine ionogram) were normal (Table 2).

**Table 2:** Laboratory investigations.

Test	Observed Value	Reference Range
Thyroid-Stimulating Hormone	1.38 mUI/L	0.4-4 mUI/L
Creatine phosphokinase	78 UI/L	10-200 UI/L
Complete Blood Count		
Hemoglobin	14 g/dL	12-15 g/dL
White Blood Cells	7.11 x 10^3^/mm³	4-10 x 10^3^ /mm³
Platelets	237x 10^3^/mm³	150-450 x 10^3^/mm³
Blood Electrolyte Panel		
Sodium levels	145 mmol/L	136-145 mmol/L
Potassium levels	2.5 mmol/L	3.6-5 mmol/L
Magnesium levels	0.95 mmol/L	0.74-1.07 mmol/L
Calcium levels	2.37 mmol/L	2.2 a 2.6 mmol/L
Phosphate levels	0.85 mmol/L	0.8 - 1.5 mmol/L
Renal Function		
Serum creatinine	6.4 mg/L	6-13 mg/L
Urea	0.23 g/L	0.18-0.45 g/L
Urinary Electrolyte Panel		
Urinary potassium excretion	50 mmol/24H	25-130 mmol/24H
Urinary sodium excretion	100 mmol/24H	55-220 mmol/24H

Whole exome sequencing revealed a heterozygous mutation in the *CACNA1S *gene, confirming the diagnosis of hypokalemic PPHy-1. The patient was treated with oral potassium supplementation during attacks and prophylactically with acetazolamide (375 mg/day), which reduced the frequency of attacks and helped avoid triggering factors. At 12 months of follow-up and treatment by acetazolamide, the patient did not experience any paralytic attacks.

## Discussion

PPHy-1 is a rare autosomal dominant channelopathy, occurring in approximately 1 in 100,000 individuals, often associated with mutations in the calcium channel CACNA1S (60%) or, less commonly, the sodium channel SCN4A (10%) [4]. The clinical presentation includes severe periodic paralytic attacks, typically resolving spontaneously. The pathogenesis is thought to involve intracellular potassium shifts, triggered by factors such as stress, post-exercise rest, or carbohydrate-rich meals. These factors stimulate catecholamine secretion, which enhances Na+/K+/ATPase pump activity, leading to paradoxical sarcolemma depolarization [5].

Inter-critical NCS can help estimate serum potassium levels during attacks using LET to guide genetic investigations. In PPHy-1, the LET typically reveals a progressive decrease in distal compound muscle action potential amplitudes, which becomes more pronounced around the 20th minute, defining pattern V, highly indicative of calcium or sodium channel mutations [6]. Our patient exhibited a pattern IV on LET, considered atypical for PPHy-1. To our knowledge, this pattern has not been documented in PPHy-1. This phenomenon may be associated with physiological processes involved in membrane repolarization post-exercise, including heightened activity of the sodium/potassium pump or increased potassium efflux [7-8].

A favorable response to acetazolamide is also characteristic of the calcium channel mutation. In the cohort of E. Matthews et al., 56% of patients with the CACNA1S mutation responded well to acetazolamide, in contrast to patients with sodium channel mutations, where improvement was seen in only 16%, and 8% even experienced worsening under acetazolamide [9]. The mechanisms underlying this response to acetazolamide in PPHy-1 with CACNA1S mutation remain unclear. It is hypothesized that acetazolamide induces non-anion gap acidosis by increasing urinary bicarbonate excretion, enhances the opening of calcium-activated potassium channels, and reduces intracellular sodium accumulation, which may decrease susceptibility to muscle paralysis [4]. We present this case to emphasize the importance of correlating clinical profiles with per-ictal serum potassium measurements in periodic paralysis, aiding in etiological investigation.

## Conclusions

This case presents an atypical electrophysiological finding in hypokalemic periodic paralysis, with pattern IV on the LET, which, to our knowledge, has not been reported in PPHy-1. Although this pattern is not typical, it suggests variability in disease expression, highlighting the importance of a comprehensive diagnostic approach. The favorable response to acetazolamide, characteristic of calcium channel mutations, underscores the need to consider mutation type in patient management. This observation calls for further investigation into the variability of electrophysiological patterns in PPHy-1 and their clinical implications.
